# Effects of a nutritional intervention using pictorial representations for promoting knowledge and practices of healthy eating among Brazilian adolescents

**DOI:** 10.1371/journal.pone.0213277

**Published:** 2019-03-11

**Authors:** Laís Gomes Fonseca, Maria Natacha Toral Bertolin, Muriel Bauermann Gubert, Eduardo Freitas da Silva

**Affiliations:** 1 Department of Nutrition, University of Brasilia, Brasilia, Federal District, Brazil; 2 Department of Statistics, University of Brasilia, Brasilia, Federal District, Brazil; The University of Hong Kong, HONG KONG

## Abstract

This study aimed to evaluate the effect of a nutritional intervention involving a problem-raising approach and the use of pictorial representations on the promotion of knowledge and practices of healthy eating among adolescents. This randomized study included 461 adolescents from public schools in Brasilia, Federal District, Brazil (intervention group: 273 students from four schools; control group: 188 students from three schools). Mean age was 14.8±1.0, and 52.9% were boys. The intervention consisted of three meetings with interactive activities about principles of healthy eating, food classification, importance of reading labels and analyzing food advertising critically, and representations of healthy and unhealthy meals and their sugar, salt, and fat content. Pictorial materials consisted of food drawings, food models, and a food packaging model. Controls were not exposed to any activity. Dietary knowledge, consumption, and behaviors were the variables of interest. The intervention group showed a higher mean score of correct answers to questions about dietary knowledge than the control group (p = 0.0006), with higher odds of correctly answering questions about *in natura* (OR: 3.7; 95% CI: 1.9–6.6), minimally processed (OR: 3.6; 95% CI: 1.9–6.4), processed (OR: 2.2; 95% CI: 1.1–4.3), and ultra-processed foods (OR: 3.5; 95% CI: 1.8–6.6) and composition of ultra-processed foods (OR: 2.4; 95% CI: 1.3–4.4). Participants in the intervention group were also 2.5 times more likely to correctly answer questions about the importance of the dietary environment (95% CI: 1.1–5.5) and caution with food advertising (95% CI: 1.2–5.3) than controls. Increased weekly consumption of vegetables (p = 0.0077; OR: 2.4; 95% CI: 1.26–4.51) and reduced consumption of soft drinks (p = 0.0212; OR: 0.36; 95% CI: 0.15–0.86) were observed in the intervention group compared to the control group. The proposed intervention increased adolescents’ knowledge and improved some of their dietary habits. Educational activities using a problem-raising approach and pictorial representations of food appear to be effective in promoting healthy eating practices among adolescents.

## Introduction

The health status of Brazilian adolescents has changed significantly in recent years, with an increasing prevalence of obesity and other comorbidities, such as hypertension and hypercholesterolemia, which were once limited to adults [1–2]. A major factor contributing to this shift is the decline in the quality of the diet of adolescents [2]. Results of national surveys show increased consumption of ultra-processed and processed foods as well as reduced intake of *in natura* and minimally processed foods among adolescents [3].

Nutrition education programs are therefore needed to raise awareness of the benefits of healthy eating. Educational approaches in the field of nutrition may vary widely [4], and only a few interventions have been successful in changing dietary habits, especially among adolescents [5]. Failure or limited impact of nutritional interventions is believed to be related to the use of non-participatory, non-stimulating methods, such as lectures and presentations [4]. In this context, the use of an active problem-raising approach may have the potential to change the eating practices of young people by stimulating reasoning about the topics and concepts presented, encouraging reflection and mainly critical thinking about their own experiences and behaviors [6].

Additionally, food- and nutrition-related activities should be particularly attractive, considering that today’s adolescents live in a computerized, dynamic, and visually attractive environment. In this respect, pictorial representations are promising tools to attract attention to health educational materials [7]. Examples of pictorial representations that may be useful in nutritional interventions include photographs of food, food models made of plastic or similar material, and images accompanied by captions or verbal guidance (pictorial cues) that are able to capture attention and arouse curiosity, stimulating reflection and discussion about the content [7– 9].

Studies on nutritional interventions using pictorial aids or materials are scarce in Brazil. In a Brazilian study of adult women, activities to promote healthy eating included the use of photographs of foods and meals. The images were used to address topics involving the amount of sugar and fat present in food, healthy food substitutions, and fruit and vegetable consumption [7]. For adolescents, a nutritional intervention to promote healthy dietary practices is currently under way in Brazil [10]. The intervention involves a digital card game that is installed in student-owned mobile devices where the cards, differentiated by color, represent healthy and unhealthy foods, meals, and dietary practices. However, the results are not yet available.

Therefore, the present study aimed to evaluate the effect of a nutritional intervention involving a problem-raising approach and the use of pictorial representations on the promotion of knowledge and practices of healthy eating among Brazilian adolescents.

## Materials and methods

This was an experimental, randomized, controlled nutritional intervention study. Written informed consent was obtained from all parents or legal guardians authorizing their children to participate in the study. All students who agreed to participate signed an assent form before inclusion in the study. The study was approved by the Research Ethics Committee of the School of Health Sciences at University of Brasilia in Brazil (approval no. 55653516.6.0000.0030).

We recruited 9th graders from public schools in Brasilia, the Federal District and capital of Brazil. In Brazil, students attending public schools are more likely to be from lower socioeconomic status families [3]. Seven schools were randomly selected by drawing lots from a sample of 20 public schools located in Brasilia. The schools were then randomly allocated to either the control group (three schools) or the intervention group (four schools). In the selected schools, all 9th graders were considered eligible and invited to participate in the study. Data were collected from September to December 2016.

The pictorial material was developed based on previous information on the composition of Brazilian adolescents’ usual meals [2, 3, 11, 12]. The pictorial representations used in this study included 46 food models, one model of retail food packaging, and nine small posters with food drawings to illustrate the dietary recommendations discussed in the meetings. To pictorially represent foods and meals, food models made of hygienic, durable, and easy-to-clean materials were purchased from stores specialized in this type of products. The models consisted of foods regularly consumed by Brazilian adolescents [11] or items of food groups recommended by the Dietary Guidelines for the Brazilian Population [13]. The food models had realistic appearance and actual size. The posters displayed the degree of food processing as well as images representing the “Ten Steps Toward Healthy Eating” according to the Dietary Guidelines for the Brazilian Population [13]. The food packaging model consisted of an enlarged version of a retail whole-grain cookie packaging and was used to support the discussion about the importance of reading labels.

The intervention consisted of three meetings with adolescents in their schools, lasting up to 45 minutes each and within an interval of 7 to 15 days. The same investigator was responsible for delivering all activities in schools in the intervention group, thus ensuring uniform exposure. The meetings consisted of interactive and participatory activities in which students had to set up meals based on their usual diet using the food models. The main goal was to establish a horizontal dialogue that would stimulate adolescents to reflect on their own dietary choices and behaviors. Several topics were discussed during the meetings, including principles of healthy eating, food classification, and the importance of reading labels and analyzing food advertising critically, as well as pictorial representations of healthy and unhealthy meals and their sugar, salt, and fat content (Table 1). Controls were not exposed to any activity.

**Table 1:** Description of topics, objectives, activities, and pictorial representations used at each meeting of the nutritional intervention. Brasilia, Federal District, Brazil, 2016.

*Topic*	*Objectives*	*Activities*
***First meeting***		
Concept of healthy eating	To reflect on diet quality; to discuss concepts of and barriers to healthy eating.	Discussion guided by questions such as “What do you think of your diet?”
Food classification	To present food classification according to the Dietary Guidelines for the Brazilian Population (2014), divided into *in natura*, minimally processed, processed, and ultra-processed foods.	Group dynamics: interactive table with food models to exemplify the four classifications and small posters with their respective names.
Food labels	To stimulate students to read and understand information on food labels; to stimulate critical thinking about food advertising.	Discussion guided by questions such as “Do you understand all the information on food packages?”
***Second meeting***		
Healthy eating	To reflect on the quality of usual breakfast and snacks; to increase the perception of sugar, salt, and fat (SSF) content in healthy and unhealthy meals; to discuss positive and negative impacts of excessive consumption of foods with high SSF content on people’s health.	Group dynamics: description of usual meals; Discussion guided by questions such as “How can your meals be improved?”; Discussion about healthy and unhealthy meals with representation of SSF content.
***Third meeting***		
Healthy eating	To discuss the importance of each meal during the day; to reflect on the importance of having complete meals at lunch and dinner; to present the principles of healthy eating (variety, balance, individuality).	Group dynamics: description of usual meals; Discussion guided by questions such as “Do you usually have a complete meal or do you have a snack for lunch and dinner?”; Discussion about healthy and unhealthy meals with representation of SSF content.

* Abbreviation: SSF: sugar, salt, and fat.

To evaluate the effects of the nutritional intervention, we used a questionnaire designed to assess dietary knowledge, consumption, and behaviors. The questionnaire was pilot tested in a previous study with adolescents from other public schools, which were not participating in the present study. A copy of the questionnaire is provided as Supporting Information available in English and Portuguese (S1 and S2 Appendices). The questionnaire was administered to all adolescents in both groups before and after the nutritional intervention, within an interval of 7 to 15 days. Demographic data, such as sex and age, were also collected during questionnaire administration. The researcher remained available for any necessary clarification while the participants were completing the questionnaires, thus ensuring that all respondents returned a complete questionnaire in both occasions.

The section of the questionnaire about dietary knowledge consisted of 13 statements that should be marked as true or false, and the total knowledge score was expressed as the number of correct answers. The following topics were addressed: principles of healthy eating (variety, balance, and moderation); nutrient composition of foods; food classification according to the degree of processing; and dietary practices (shopping at farmer’s markets, having meals at adequate environments, time spent having meals, and reading food labels critically). This section was developed based on the concepts of the Dietary Guidelines for the Brazilian Population [13], with a qualitative approach that differs significantly from previous Brazilian studies that have evaluated dietary knowledge based on strictly biological aspects related to food composition and number of servings recommended per food group [14,15].

A validated questionnaire used in the National School-Aged Adolescent Health Survey was adapted to develop the sections on dietary consumption and behavior [16]. Data were collected for consumption of healthy eating markers (beans, vegetables, fruits, and milk) and unhealthy eating markers (soft drinks, French fries, deep-fried snacks, sweets, cold cuts, cookies, and crackers) in the previous 7 days. Regular consumption was defined as the consumption of a food item at least five times in the previous 7 days. The dietary behavior section included questions about the frequency of having lunch or dinner with parents or caregivers, eating while studying or watching TV, and having breakfast. This section also contained questions for self-perceived knowledge of healthy eating and diet quality. To this end, participants were asked “How much do you know about healthy eating?” and “Do you consider your diet healthy?”. They should assign a score from 0 to 10 to each item, where 0 = “I don’t know anything about this” and “It’s not healthy at all”, respectively, and 10 = “I know a lot about healthy eating” and “It’s very healthy”, respectively.

To detect an effect size of 0.10 between the null hypothesis of equal means between groups and the alternative hypothesis of different means between groups, with a significance level of 5%, a power of 80%, and a correlation of 0.71 between observations at two occasions, a sample size of 455 adolescents was necessary to be divided into intervention and control groups.

The effect of the intervention was assessed by comparing (a) correct answers to items about dietary knowledge, (b) regular dietary consumption, (c) regular dietary behaviors, and (d) scores for self-perceived knowledge of healthy eating and diet quality between the two groups, before and after the intervention. For regular dietary consumption and behavior, a generalized estimating equation (GEE) model with binary logit function was used. Odds ratios (OR) and respective confidence intervals (95% CI) were calculated. The mean scores for correct answers to questions about dietary knowledge, self-perceived knowledge of healthy eating, and self-perceived diet quality were compared between groups using an analysis of covariance (ANCOVA) model. In the GEE and ANCOVA models, post-intervention measures were considered dependent variables, group (intervention or control) was considered an independent variable, and pre-intervention measures and attendance to all meetings were considered covariates. Statistical significance was set at p < 0.05. The analyses were performed using SAS, version 9.4 (SAS Institute, Inc., 2012).

## Results

The initial sample consisted of 676 adolescents, and 31.8% were lost before the end of the study. Losses were mainly due to the absence of students on the second occasion of questionnaire administration. Only those who completed the questionnaire in both occasions (pre- and post-intervention) were included in the analyses, resulting in a final sample of 461 adolescents. Of these, 273 students were in the intervention group and 188 in the control group. The mean age of participants was 14.8 ± 1.0 years, and 52.9% were boys, with no significant difference between groups.

As for the effects of the intervention on dietary knowledge, the intervention group, at the end of the study, showed a significantly higher mean score of correct answers than the control group (p < 0.001) (Table 2).

**Table 2:** Mean score of correct answers to questions about dietary knowledge before and after the intervention. Brasilia, Federal District, Brazil, 2016.

Mean score of correct answers to questions about dietary knowledge	Study group Mean (95% CI)		Post-intervention between-group difference Mean (95% CI)	p-value
	Intervention	Control		
Pre-intervention	7.54 (7.20–7.88)	7.19 (6.78–7.59)		
Post-intervention	9.26 (8.91–9.61)	7.19 (6.77–7.61)		
Post-intervention (adjusted)*	8.95 (8.61–9.28)	7.77 (7.29–8.24)	-1.18 (-1.85–0.51)	0.001

* Data adjusted for baseline values and attendance to all meetings as covariates. Abbreviation: 95% CI: 95% confidence interval.

The intervention group showed higher odds of correctly answering questions about food processing than the control group, including questions about *in natura* foods (OR: 3.7; 95% CI: 1.9–6.6), minimally processed foods (OR: 3.6; 95% CI: 1.9–6.6), processed foods (OR: 2.2; 95% CI: 1.1–4.3), and ultra-processed foods (OR: 3.5; 95% CI:1.8–6.6). In addition, participants in the intervention group were 2.5 times more likely to correctly answer questions about the importance of the dietary environment (95% CI: 1.1–5.5) and the reliability of information from food advertising (95% CI: 1.2–5.3) than controls (Table 3).

**Table 3:** Distribution of correct answers in the questionnaire about knowledge of healthy eating before and after the intervention, with odds ratio of answering the items correctly after the proposed intervention. Brasilia, Federal District, Brazil, 2016.

Questionnaire items (*answers*)	Intervention group				Control group				Post-intervention between-group comparison	
	Before		After		Before		After			
	n	%	n	%	n	%	n	%	OR (95% CI)	p-value
Healthy eating means following a strict diet (*false*)	183	71.4	175	68.3	124	66.3	129	68.9	0.6 (0.3–1.3)	0.217
Healthy eating means eating various foods moderately (*true*)	208	81.8	233	91.7	139	74.3	145	77.5	1.8 (0.7–4.3)	0.185
A balanced diet may include sweets (*true*)	186	75.0	203	81.8	121	65.4	133	71.8	0.8 (0.4–1.8)	0.734
Sandwich cookies contain low fat and high sugar content (*false*)	81	31.4	138	53.4	60	32.9	62	33.1	2.4 (1.3–4.4)	0.004
Beverages such as juice boxes contain low sugar and high fruit content (*false*)	221	85.3	227	87.6	150	80.6	139	74.7	1.4 (0.7–3.1)	0.325
*In natura* foods should be the basis of our diet (*true*)	90	35.1	191	74.6	59	30.5	58	31.0	3.7 (1.9–6.6)	< 0.001
Fruits and vegetables may be options of minimally processed foods (*true*)	81	31.6	173	67.5	75	40.3	73	39.2	3.6 (1.9–6.4)	< 0.001
The ingredients and methods used in food processing make food less healthy (*true*)	181	70.1	194	75.1	114	60.9	103	55.0	2.2 (1.1–4.3)	0.023
Ultra-processed foods are healthier than minimally processed foods (*false*)	131	50.7	208	80.6	85	45.4	81	43.3	3.5 (1.8–6.6)	< 0.001
We should avoid shopping at farmer’s markets because they have few options of healthy foods (*false*)	208	81.9	221	83.0	134	72.0	134	72.0	1.3 (0.6–2.8)	0.409
Home cooking is a healthy practice because you can use several frozen foods and ready-to-use seasoning mixes (*false*)	62	24.1	69	26.8	41	21.9	38	20.3	1.5 (0.8–3.0)	0.184
Lack of time, space, and company may influence diet quality (*true*)	203	79.3	200	78.1	131	70.0	126	67.3	2.5 (1.1–5.5)	0.026
In general, information, instructions, and messages from TV food advertising are reliable and thus we can believe them (*false*)	183	71.4	203	79.3	118	63.1	122	65.2	2.5 (1.2–5.3)	0.013

Abbreviations: OR: odds ratio; 95% CI: 95% confidence interval.

Regarding dietary consumption, weekly consumption of raw or cooked vegetables increased among participants in the intervention group, who were 2.4 times more likely to eat such foods (p < 0.001; 95% CI: 1.26–4.51) and 64% less likely to consume soft drinks regularly (p = 0.021; OR = 0.36; 95% CI: 0.15–0.86) than controls. No significant post-intervention difference was found between groups in weekly consumption of other food items (Table 4).

**Table 4:** Regular dietary consumption of food items* before and after the intervention, with odds ratio of changing behavior after the proposed intervention. Brasilia, Federal District, Brazil, 2016.

Food items	Intervention group				Control group				Post-intervention between-group comparison	
	Before		After		Before		After			
	n	%	n	%	n	%	n	%	OR (95% CI)	p-value
Beans^†^	194	76.6	185	73.1	119	69.2	120	69.7	1.29 (0.50–3.30)	0.560
Deep-fried snacks^‡^	25	9.4	29	10.9	16	8.7	20	20.2	0.41 (0.12–1.36)	0.143
Cold cuts^‡^	36	13.5	30	11.2	24	12.7	21	11.2	0.40 (0.11–1.44)	0.161
Raw/cooked vegetables^†^	93	34.8	105	39.3	68	36.1	44	23.4	2.38 (1.26–4.51)	0.007
Raw salad^†^	104	38.9	112	41.9	61	32.6	53	28.3	1.50 (0.78–2.90)	0.222
Cooked vegetables^†^	42	15.6	47	17.4	29	15.4	20	10.6	1.45 (0.61–3.46)	0.400
Crackers^‡^	73	27.2	52	19.4	60	32.2	43	23.1	0.44 (0.18–1.10)	0.079
Cookies^‡^	62	23.6	54	20.6	60	33.3	39	21.6	1.79 (0.36–1.74)	0.560
Packaged snacks^‡^	21	7.9	19	7.2	24	13.1	22	12.0	0.80 (0.28–2.24)	0.672
Sweets^‡^	90	33.9	77	29.0	75	40.3	61	32.8	0.55 (0.27–1.13)	0.103
Fresh fruits^†^	96	36.0	93	34.9	51	28.1	43	23.7	1.17 (0.60–2.29)	0.642
Milk^†^	151	56.3	135	50.3	108	58.6	80	43.0	1.88 (0.97–3.67)	0.063
Soft drinks^‡^	84	31.2	61	22.6	60	31.9	54	28.7	0.36 (0.15–0.86)	0.021

* Regular consumption was defined as the consumption of a food item at least five times in the previous 7 days.

† Healthy eating markers.

‡ Unhealthy eating markers. Abbreviations: OR: odds ratio; 95% CI: 95% confidence interval.

The intervention produced no change in behaviors such as having lunch or dinner with the family, eating while watching TV or studying, or eating breakfast (Table 5).

**Table 5:** Dietary behaviors before and after the intervention, with odds ratio of changing behavior after the proposed intervention. Brasilia, Federal District, Brazil, 2016.

Dietary behaviors	Intervention group				Control group				Post-intervention between-group comparison	
	Before		After		Before		After			
	n	%	n	%	n	%	n	%	OR (95% CI)	p-value
Eating with parents	145	83.8	139	80.3	105	82.0	99	77.3	1.86 (0.50–6.89)	0.350
Eating while watching TV/studying	125	77.1	130	80.2	101	79.5	99	77.9	0.81 (0.19–3.51)	0.780
Eating breakfast	142	76.7	143	77.3	100	73.5	103	75.7	2.67 (0.68–7.57)	0.182

Abbreviations: OR: odds ratio; 95% CI: 95% confidence interval.

No significant changes were observed in the scores for self-perceived knowledge of healthy eating and diet quality (Table 6).

**Table 6:** Scores for self-perceived knowledge of healthy eating and diet quality before and after the intervention. Brasilia, Federal District, Brazil, 2016.

	Between-group comparisons Mean (95% CI)		Post-intervention between-group difference	p-value
	Intervention group	Control group	Mean (95% CI)	
Score for self-perceived knowledge of healthy eating:				
Pre-intervention	6.47 (6.20–6.75)	6.41 (6.03–6.80)		
Post-intervention	6.95 (6.70–7.20)	6.26 (5.90–6.62)		
Post-intervention (adjusted)*	6.82 (6.55–7.10)	6.46 (6.07–6.85)	-0.36 (-0.92–0.19)	0.199
Score for self-perceived diet quality:				
Pre-intervention	5.98 (5.76–6.21)	5.70 (5.37–6.02)		
Post-intervention	6.21 (5.98–6.44)	6.02 (5.70–6.35)		
Post-intervention (adjusted)*	6.11 (5.87–6.33)	6.16 (5.83–6.49)	-0.06 (-0.41–0.53)	0.909

* Data adjusted for baseline values and attendance to all meetings as covariates. Abbreviation: 95% CI: 95% confidence interval.

## Discussion

This study showed that a short-term nutritional intervention based on an active, participatory, problem-raising approach with the use of pictorial representations of food was able to increase dietary knowledge among adolescents. The intervention also had some influence on their dietary consumption, leading to increased vegetable consumption and reduced soft drink intake.

The proposed intervention was successful in increasing nutritional knowledge, especially by enhancing the understanding of food classification according to the degree of food processing. The Dietary Guidelines for the Brazilian Population, officially adopted in 2006 and updated in 2014, are based on an innovative dietary approach focusing on qualitative dietary intake rather than on the traditional recommendations of number of servings per food group, thereby stimulating the consumption of *in natura* and minimally processed foods and restricting the consumption of processed and ultra-processed foods [13]. The positive findings in our study indicate that adolescents were able to more adequately recognize unhealthy products, whose excessive consumption is associated with several morbidities. However, the deleterious effects of the global food system have become apparent in the diet of Brazilians, especially in adolescents, despite Brazil’s healthy food culture [17,18]. Studies have shown that many Brazilian adolescents are either unaware of or do not use traditional native foods [12, 19, 20]. The consumption of processed and ultra-processed foods, in turn, has increased substantially, contributing to the current obesity epidemic [21].

Robinson et al. showed that eating in inappropriate places favors distraction, leading to increased energy consumption [22]. However, this is an increasingly common practice among adolescents, as they often have meals while watching TV or using electronic devices, including high consumption of energy-dense foods [23,24]. This practice negatively affects the perceptions of taste and satiety, leading to an increased need to eat snacks during the day [18, 25, 26]. Our results suggest that participants began to recognize that factors such as availability of time to prepare a meal and the environment where they eat are important for diet quality, although they have not changed behaviors such as eating in front of the TV.

The fact that significant changes were not obtained in the behaviors under study is not surprising. Producing changes in complex behaviors, such as food habits, requires more intensive long-term interventions. In addition, we cannot overlook the fact that eating while studying or watching TV as well as skipping meals are common practices among adolescents. These behaviors are conceptualized as food habits or practices embedded in social and family life, and therefore are not expected to be modified only by increasing knowledge about them [27].

Our results also showed that exposure to the intervention increased adolescents’ ability to recognize that food advertising is not always reliable and that information from food labels should be read with attention. Media exerts a direct influence on dietary preferences and choices of children and adolescents, contributing to the increasing consumption of processed and ultra-processed foods and, consequently, to a greater risk of obesity in this population [28]. Therefore, there is a clear need for actions to protect young people against the means that stimulate consumption of unhealthy products [25]. Countries such as England, Australia and Norway have prohibited advertising and marketing to children under the age of 16, 14 and 12 years, respectively [29]. In Brazil, current legislation does not prohibit unhealthy food advertising to adolescents. Although children under the age of 2 years are protected by the Brazilian Standard of Food Marketing to Infants and Young Children, there is still a long way to go when it comes to children and adolescents [30].

The nutritional intervention also produced some favorable changes in dietary consumption among adolescents, such as increased vegetable consumption and reduced soft drink intake. At about 15 years of age, adolescents have a considerable degree of autonomy to make their dietary choices, hence the importance of interventions that alert and sensitize adolescents to these choices in order to make them conscious consumers [31, 32]. Recent studies have obtained similar results. Price et al. [33] promoted practical cooking activities for American adolescents and observed that 71.2% reported eating more vegetables and 73% reported drinking less soda/soft drinks.

The use of pictorial representations as part of the educational activity was particularly relevant for the positive results obtained in the present study. Other authors using images or photographs in nutritional interventions have also obtained positive results in different populations [7, 8, 34]. In a review of studies of food and nutrition education for Brazilian students, Ramos et al. [4] highlighted the frequent use of non-participatory activities, exposing a gap between theory and practice as well as a disregard for the subjectivity involved in dietary choices. Toral et al. [35] concluded that innovative strategies should be used to motivate adolescents to make healthy food choices, based on the results of their study showing that the use of printed educational material alone was insufficient to increase fruit and vegetable consumption in this population.

The limitations of the present study include a substantial loss of participants and short duration of the intervention to change dietary behaviors. The low retention rate may be attributed to a bus drivers’ strike that occurred in the city during the second occasion of questionnaire administration, preventing many students from going to school. According to Murimi et al. [36] and Ramos et al. [4], long-term nutritional interventions (i.e., more than 1 year) and school community and family engagement are recommended to improve dietary habits and nutritional status among young students. We acknowledge that an intervention with more than three meetings would probably yield more significant results. However, important results were achieved with the activities used in our intervention, despite their short-term focus, with a positive impact on adolescent dietary intake. Furthermore, the investigators considered that adolescents have a considerable degree of autonomy to make their dietary choices, which justifies the non-engagement of family or school. Another limitation is the lack of a long-term evaluation of the maintenance of results, which should be conducted in future studies, as well as the effects of the intervention on adolescents of different socioeconomic status, as the study was limited to students from public schools.

In conclusion, nutritional intervention involving educational activities based on a problem-raising approach and the use of pictorial representations of food appears to be effective in promoting healthy eating practices among adolescents. Further research is needed to assess whether the proposed model is more effective than traditional interventions, which do not use a problem-raising approach or pictorial representations. Because the control group of this study was not exposed to any activity, this aspect could not be evaluated.

## Supporting information

S1 Appendix Questionnaire used in the study (English). Questionnaire applied to Brazilian adolescents to assess dietary knowledge, consumption, and behaviors, as well as demographic data.
(DOC)

S2 Appendix Questionnaire used in the study (Portuguese). Questionnaire applied to Brazilian adolescents to assess dietary knowledge, consumption, and behaviors, as well as demographic data.
(DOCX)

S1 Data Intervention data. Archive with demographic information of the study subjects and measurements of dietary knowledge, dietary intake and eating behaviors for each of the two groups (control and intervention) before and after the nutritional intervention.
(XLSX)
