# Supplementary material for: Effects of a nutritional intervention using pictorial representations for promoting knowledge and practices of healthy eating among Brazilian adolescents
Source: PLoS One. 2019 Mar 11;14(3):e0213277. doi: 10.1371/journal.pone.0213277 (PMC6411163; doi:10.1371/journal.pone.0213277)
Supplement: S1 Appendix — Questionnaire applied to Brazilian adolescents to assess dietary knowledge, consumption, and behaviors, as well as demographic data. (DOC) [file pone.0213277.s001.doc]

STUDENT'S IDENTIFICATION

1. What is your name? ____________________________________________________
2. Where do you study? ____________________________________________________
3. Which grade/class are you in? ______________________________________________
4. When does your school day start? 1. ( ) Morning 2. ( ) Afternoon
5. What is your sex? 1. ( ) Male 2. ( ) Female
6. How old are you? ______years

**The next questions refer to your diet. Take into account all you have eaten at home, at school, at snack bars, at restaurants, or at any other place. (Mark an X on your answer)**

13) Which of your yesterday's meals included raw salad? Examples: lettuce, tomato, carrot, cucumber, onion, etc.

1. ( ) I didn't eat raw salad yesterday
2. ( ) Yesterday's lunch
3. ( ) Yesterday's dinner
4. ( ) Yesterday's lunch and dinner

14) Which of your yesterday's meals included cooked vegetables, excluding potato and manioc (cassava)?

1. ( ) I didn't eat cooked vegetables yesterday
2. ( ) Yesterday's lunch
3. ( ) Yesterday's dinner
4. ( ) Yesterday's lunch and dinner

15) How many times did you eat fresh fruits yesterday?

1. ( ) I didn't eat fresh fruits yesterday
2. ( ) Once yesterday
3. ( ) Twice yesterday
4. ( ) Three times or more yesterday

| **IN THE PAST 7 DAYS, how frequently did you eat/drink the following items? (Mark an X on your answer)** | | | | | | | | | |
| --- | --- | --- | --- | --- | --- | --- | --- | --- | --- |
|  | **Food item(s)** | **Consumption frequency in the PAST 7 DAYS** | | | | | | | |
| 16) | Beans | Not at all | 1 day | 2 days | 3 days | 4 days | 5 days | 6 days | Every day |
| 17) | Deep-fried snacks. Examples: French fries (excluding packaged potato chips), chicken croquette, kibbe, *pastel*, *acarajé*, etc. | Not at all | 1 day | 2 days | 3 days | 4 days | 5 days | 6 days | Every day |
| 18) | Hamburger, sausage, mortadella, salami, ham, nuggets | Not at all | 1 day | 2 days | 3 days | 4 days | 5 days | 6 days | Every day |
| 19) | At least one type of raw or cooked vegetable. Examples: cabbage, tomato, lettuce, squash, chayote, broccoli, spinach, etc. Exclude potato and manioc (cassava). | Not at all | 1 day | 2 days | 3 days | 4 days | 5 days | 6 days | Every day |
| 20) | Raw salad. Examples: lettuce, tomato, carrot, cucumber, onion, etc. | Not at all | 1 day | 2 days | 3 days | 4 days | 5 days | 6 days | Every day |
| 21) | Cooked vegetables in a meal, including soup. Examples: cabbage, squash, chayote, broccoli, spinach, carrot, etc. Exclude potato and manioc (cassava). | Not at all | 1 day | 2 days | 3 days | 4 days | 5 days | 6 days | Every day |
| 22) | Crackers | Not at all | 1 day | 2 days | 3 days | 4 days | 5 days | 6 days | Every day |
| 23) | Cookies | Not at all | 1 day | 2 days | 3 days | 4 days | 5 days | 6 days | Every day |
| 24) | Packaged fried snacks (including potato chips) | Not at all | 1 day | 2 days | 3 days | 4 days | 5 days | 6 days | Every day |
| 25) | Sweets (candy, chocolate, gum, lollipops) | Not at all | 1 day | 2 days | 3 days | 4 days | 5 days | 6 days | Every day |
| 26) | Fresh fruits or fruit salad | Not at all | 1 day | 2 days | 3 days | 4 days | 5 days | 6 days | Every day |
| 27) | Milk (exclude soy milk and consider, for example, milk with coffee or chocolate powder, smoothie, porridge) | Not at all | 1 day | 2 days | 3 days | 4 days | 5 days | 6 days | Every day |
| 28) | Soft drinks | Not at all | 1 day | 2 days | 3 days | 4 days | 5 days | 6 days | Every day |

1. Do you usually have lunch or dinner with your mother, father, or guardian?
2. ( ) No
3. ( ) Yes, every day
4. ( ) Yes, 5 to 6 times a week
5. ( ) Yes, 3 to 4 times a week
6. ( ) Yes, 1 to 2 times a week
7. ( ) Yes, but rarely
8. Do you usually eat while watching TV or studying?
9. ( ) No
10. ( ) Yes, every day
11. ( ) Yes, 5 to 6 times a week
12. ( ) Yes, 3 to 4 times a week
13. ( ) Yes, 1 to 2 times a week
14. ( ) Yes, but rarely
15. Do you usually have breakfast?
16. ( ) No
17. ( ) Yes, every day
18. ( ) Yes, 5 to 6 times a week
19. ( ) Yes, 3 to 4 times a week
20. ( ) Yes, 1 to 2 times a week
21. ( ) Yes, but rarely
22. **Do you consider your diet healthy?** With that in mind, how would you grade your diet, being 0 = “It’s not healthy at all” & 10 = “It’s very healthy”? _____
23. **How much do you know about healthy eating?** With that in mind, how would you grade your knowledge, being 0 = “I don’t know anything about this” & 10 = "I know a lot about healthy eating”? _____

| **KNOWLEDGE QUESTIONNAIRE** | | | | |
| --- | --- | --- | --- | --- |
| 34) | Healthy eating means following a strict diet. | True | **False** | I don't know |
| 35) | Healthy eating means eating various foods moderately. | **True** | False | I don't know |
| 36) | A balanced diet may include sweets. | **True** | False | I don't know |
| 37) | Sandwich cookies contain low fat and high sugar content. | True | **False** | I don't know |
| 38) | Beverages such as juice boxes contain low sugar and high fruit content. | True | **False** | I don't know |
| 39) | *In natura*foods should be the basis of our diet. | **True** | False | I don't know |
| 40) | Fruits and vegetables may be options of minimally processed foods. | **True** | False | I don't know |
| 41) | The ingredients and methods used in food processing make food less healthy (e.g., canned vegetables). | **True** | False | I don't know |
| 42) | Ultra-processed foods are healthier than minimally processed foods. | True | **False** | I don't know |
| 43) | We should avoid shopping at farmer’s markets because they have few options of healthy foods. | True | **False** | I don't know |
| 44) | Home cooking is a healthy practice because you can use several frozen foods and ready-to-use seasoning mixes. | True | **False** | I don't know |
| 45) | Lack of time, space, and company may influence diet quality. | **True** | False | I don't know |
| 46) | In general, information, instructions, and messages from TV food advertising are reliable and thus we can believe them. | True | **False** | I don't know |
