# Supplementary material for: Effects of a nutritional intervention using pictorial representations for promoting knowledge and practices of healthy eating among Brazilian adolescents
Source: PLoS One. 2019 Mar 11;14(3):e0213277. doi: 10.1371/journal.pone.0213277 (PMC6411163; doi:10.1371/journal.pone.0213277)
Supplement: S2 Appendix — Questionnaire applied to Brazilian adolescents to assess dietary knowledge, consumption, and behaviors, as well as demographic data. (DOCX) [file pone.0213277.s002.docx]

IDENTIFICAÇÃO DO ALUNO

1. Qual é o seu nome? ____________________________________________________
2. Qual é a sua escola? ____________________________________________________
3. Qual é a sua Série/ Turma? ______________________________________________
4. Qual período você estuda? 1. ( ) Manhã 2. ( ) Tarde
5. Qual é o seu sexo? 1. ( ) Masculino 2. ( ) Feminino
6. Qual é a sua idade? ______anos

**As próximas perguntas referem-se a sua alimentação. Leve em conta tudo o que você comeu em casa, na escola, na rua, em lanchonetes, em restaurantes ou em qualquer outro lugar. (Marque um X na sua resposta)**

13) Ontem, em quais refeições você comeu salada crua? Exemplos: alface, tomate, cenoura, pepino, cebola, etc.

1. ( ) Não comi salada crua ontem
2. ( ) No almoço de ontem
3. ( ) No jantar de ontem
4. ( ) No almoço e no jantar de ontem

14) Ontem, em quais refeições você comeu legumes ou verduras cozidas, sem contar batata e aipim (mandioca/macaxeira)?

1. ( ) Não comi legumes nem verduras cozidos ontem
2. ( ) No almoço de ontem
3. ( ) No jantar de ontem
4. ( ) No almoço e no jantar de ontem

15) Ontem, quantas vezes você comeu frutas frescas?

1. ( ) Não comi frutas frescas ontem
2. ( ) Uma vez ontem
3. ( ) Duas vezes ontem
4. ( ) Três vezes ou mais ontem

| **NOS ÚLTIMOS 7 DIAS, em quantos dias você comeu/bebeu os seguintes alimentos? (Marque um X na sua resposta)** | | | | | | | | | |
| --- | --- | --- | --- | --- | --- | --- | --- | --- | --- |
|  | **Alimento (s)** | **Frequência do consumo nos ÚLTIMOS 7 DIAS** | | | | | | | |
| 16) | Feijão | Não comi | 1 dia | 2 dias | 3 dias | 4 dias | 5 dias | 6 dias | Todos os dias |
| 17) | Salgados frito. Exemplo: batata frita (sem contar a batata de pacote) ou salgados fritos como coxinha de galinha, quibe frito, pastel frito, acarajé, etc. | Não comi | 1 dia | 2 dias | 3 dias | 4 dias | 5 dias | 6 dias | Todos os dias |
| 18) | Hambúrguer, salsicha, mortadela, salame, presunto, *nuggets* ou linguiça | Não comi | 1 dia | 2 dias | 3 dias | 4 dias | 5 dias | 6 dias | Todos os dias |
| 19) | Pelo menos um tipo de legume ou verdura crus ou cozidos. Exemplo: couve, tomate, alface, abóbora, chuchu, brócolis, espinafre, etc. Não inclua batata e aipim (mandioca/macaxeira). | Não comi | 1 dia | 2 dias | 3 dias | 4 dias | 5 dias | 6 dias | Todos os dias |
| 20) | Salada crua. Exemplo: alface, tomate, cenoura, pepino, cebola, etc. | Não comi | 1 dia | 2 dias | 3 dias | 4 dias | 5 dias | 6 dias | Todos os dias |
| 21) | Legumes ou verduras cozidos na comida, inclusive sopa? Exemplo: couve, abóbora, chuchu, brócolis, espinafre, cenoura, etc. Não inclua batata e aipim (mandioca/macaxeira). | Não comi | 1 dia | 2 dias | 3 dias | 4 dias | 5 dias | 6 dias | Todos os dias |
| 22) | Biscoitos salgados ou bolachas salgadas | Não comi | 1 dia | 2 dias | 3 dias | 4 dias | 5 dias | 6 dias | Todos os dias |
| 23) | Biscoitos doces ou bolachas doces | Não comi | 1 dia | 2 dias | 3 dias | 4 dias | 5 dias | 6 dias | Todos os dias |
| 24) | Salgadinho de pacote ou batata frita de pacote | Não comi | 1 dia | 2 dias | 3 dias | 4 dias | 5 dias | 6 dias | Todos os dias |
| 25) | Guloseimas (doces, balas, chocolates, chicletes, bombons ou pirulitos) | Não comi | 1 dia | 2 dias | 3 dias | 4 dias | 5 dias | 6 dias | Todos os dias |
| 26) | Frutas frescas ou salada de frutas | Não comi | 1 dia | 2 dias | 3 dias | 4 dias | 5 dias | 6 dias | Todos os dias |
| 27) | Leite (Excluir “leite” de soja e considerar, por exemplo, leite com café ou chocolate, vitamina, mingau) | Não comi | 1 dia | 2 dias | 3 dias | 4 dias | 5 dias | 6 dias | Todos os dias |
| 28) | Refrigerante | Não comi | 1 dia | 2 dias | 3 dias | 4 dias | 5 dias | 6 dias | Todos os dias |

1. Você costuma almoçar ou jantar - com sua mãe, pai ou responsável?
2. ( ) Não
3. ( ) Sim, todos os dias
4. ( ) Sim, 5 a 6 dias por semana
5. ( ) Sim, 3 a 4 dias por semana
6. ( ) Sim, 1 a 2 dias por semana
7. ( ) Sim, mas apenas raramente
8. Você costuma comer quando está assistindo à TV ou estudando?
9. ( ) Não
10. ( ) Sim, todos os dias
11. ( ) Sim, 5 a 6 dias por semana
12. ( ) Sim, 3 a 4 dias por semana
13. ( ) Sim, 1 a 2 dias por semana
14. ( ) Sim, mas apenas raramente
15. Você costuma tomar o café da manhã?
16. ( ) Não
17. ( ) Sim, todos os dias
18. ( ) Sim, 5 a 6 dias por semana
19. ( ) Sim, 3 a 4 dias por semana
20. ( ) Sim, 1 a 2 dias por semana
21. ( ) Sim, mas apenas raramente
22. **Você considera sua alimentação saudável?** Pensando nisso, que nota você daria para sua alimentação, sendo 0 nada saudável e 10 muito saudável? _____
23. **Quanto você sabe sobre alimentação saudável?** Pensando nisso, que nota você daria para seu conhecimento, sendo 0 não sei nada e 10 sei muito sobre alimentação saudável? ____

| **QUESTIONÁRIO DE CONHECIMENTO** | | | | |
| --- | --- | --- | --- | --- |
| 34) | Alimentação saudável significa seguir uma dieta. | Verdadeiro | **Falso** | Eu não sei |
| 35) | Alimentação saudável significa comer diversos alimentos de forma moderada. | **Verdadeiro** | Falso | Eu não sei |
| 36) | Em uma dieta equilibrada, é possível consumir doces. | **Verdadeiro** | Falso | Eu não sei |
| 37) | Bolacha recheada contém pouca gordura e muito açúcar. | Verdadeiro | **Falso** | Eu não sei |
| 38) | Bebidas como suco de caixinha contém pouco açúcar e muita fruta. | Verdadeiro | **Falso** | Eu não sei |
| 39) | Os alimentos *in natura* devem ser a base da nossa alimentação. | **Verdadeiro** | Falso | Eu não sei |
| 40) | Frutas e verduras podem ser opções de alimentos minimamente processados. | **Verdadeiro** | Falso | Eu não sei |
| 41) | Os ingredientes e métodos usados na fabricação de alimentos processados tornam os alimentos menos saudáveis (Ex. conserva de legumes). | **Verdadeiro** | Falso | Eu não sei |
| 42) | Alimentos ultraprocessados são mais saudáveis que os alimentos minimamente processados. | Verdadeiro | **Falso** | Eu não sei |
| 43) | Devemos evitar fazer compras em feiras livres, pois as feiras têm poucas opções de alimentos saudáveis. | Verdadeiro | **Falso** | Eu não sei |
| 44) | Cozinhar em casa é uma prática considerada saudável, pois existem várias opções de alimentos congelados e temperos prontos. | Verdadeiro | **Falso** | Eu não sei |
| 45) | A falta de tempo, o ambiente e a companhia podem influenciar na qualidade da alimentação. | **Verdadeiro** | Falso | Eu não sei |
| 46) | De forma geral, as informações, orientações e mensagens das propagandas de alimentos que passam na TV são confiáveis e, portanto, podemos acreditar nelas. | Verdadeiro | **Falso** | Eu não sei |
